# Functional and phylogenetic diversity of woody plants drive herbivory in a highly diverse forest

**DOI:** 10.1111/nph.12695

**Published:** 2014-01-24

**Authors:** Andreas Schuldt, Thorsten Assmann, Helge Bruelheide, Walter Durka, David Eichenberg, Werner Härdtle, Wenzel Kröber, Stefan G Michalski, Oliver Purschke

**Affiliations:** 1 Institute of Ecology, Leuphana University LüneburgScharnhorststr. 1, D-21335, Lüneburg, Germany; 2 Institute of Biology/Geobotany and Botanical Garden, University of HalleAm Kirchtor 1, D-06108, Halle, Germany; 3 German Centre for Integrative Biodiversity Research (iDiv) Halle-Jena-LeipzigDeutscher Platz 5e, D-04103, Leipzig, Germany; 4 Department of Community Ecology, Helmholtz Centre for Environmental Research – UFZTheodor-Lieser-Str. 4, D-06120, Halle, Germany

**Keywords:** BEF-China, biodiversity, ecosystem functioning, functional traits, negative density dependence, plant–insect interactions, species richness

## Abstract

1. Biodiversity loss may alter ecosystem processes, such as herbivory, a key driver of ecological functions in species-rich (sub)tropical forests. However, the mechanisms underlying such biodiversity effects remain poorly explored, as mostly effects of species richness – a very basic biodiversity measure – have been studied. Here, we analyze to what extent the functional and phylogenetic diversity of woody plant communities affect herbivory along a diversity gradient in a subtropical forest.
2. We assessed the relative effects of morphological and chemical leaf traits and of plant phylogenetic diversity on individual-level variation in herbivory of dominant woody plant species across 27 forest stands in south-east China.
3. Individual-level variation in herbivory was best explained by multivariate, community-level diversity of leaf chemical traits, in combination with community-weighted means of single traits and species-specific phylodiversity measures. These findings deviate from those based solely on trait variation within individual species.
4. Our results indicate a strong impact of generalist herbivores and highlight the need to assess food-web specialization to determine the direction of biodiversity effects. With increasing plant species loss, but particularly with the concomitant loss of functional and phylogenetic diversity in these forests, the impact of herbivores will probably decrease – with consequences for the herbivore-mediated regulation of ecosystem functions.

## Introduction

The realization that global change alters the biotic composition of ecosystems has spawned a wealth of research showing that biodiversity loss affects significantly ecosystem functions and services (Cardinale *et al*., 2012; Naeem *et al*., 2012). However, our understanding of the mechanisms underlying observed diversity effects is still limited, as many studies have focused on species richness as a very basic measure of biodiversity (Hillebrand & Matthiessen, 2009). More recently, the awareness that the functional traits of species (e.g. morphological or physiological features that determine an organism’s performance) play a central role in the determination of many of these diversity effects has led to a stronger focus on the functional dimensions of biodiversity and a more thorough investigation into the role of specific traits for individual functions (Diaz *et al*., 2007; Reiss *et al*., 2009). However, although progress in our understanding of functional diversity effects has been made, particularly for processes within single trophic levels (primarily the producer level), it is increasingly being recognized that, in many cases, trophic interactions are key modifiers of these relationships (Reiss *et al*., 2009; Cardinale *et al*., 2012). Herbivory may be particularly crucial in this respect.

Herbivory strongly influences nutrient cycles, productivity and the diversity maintenance of ecosystems (Schmitz, 2008; Schowalter, 2012; Terborgh, 2012). Moreover, the strength of herbivory effects has been shown to vary with plant diversity (Jactel & Brockerhoff, 2007; Schuldt *et al*., 2010; Cardinale *et al*., 2012). However, we still lack a mechanistic understanding of the relationship between herbivory and plant diversity. Some plant traits commonly assumed to determine levels of herbivory within and among species, such as secondary metabolites, have been found to perform poorly in predicting overall damage levels under natural conditions (Carmona *et al*., 2011; Schuldt *et al*., 2012; see also Paine *et al*., 2012), and the general pattern seems to be that several traits act in combination to make a plant attractive to herbivores or to repel them (Agrawal & Fishbein, 2006; Loranger *et al*., 2012). Multivariate trait indices or even an estimation of functional trait space by phylogenetic diversity (Srivastava *et al*., 2012) might thus be stronger predictors than single traits. Phylogenetic diversity incorporates the evolutionary history of species relationships and may thus not only capture phylogenetically conserved dissimilarity of (often unmeasured) traits among species. It also indicates shared evolutionary relationships between herbivores and their host plants (Cavender-Bares *et al*., 2009; Srivastava *et al*., 2012), and has been shown to predict herbivory-induced seedling mortality in some cases better than the diversity of functional traits commonly considered to be important for herbivores (Paine *et al*., 2012). Moreover, non-additive effects of increasing plant species richness on herbivory patterns indicate that not only the traits of a focal plant species, but also community properties, play an important role in determining herbivore damage levels (Loranger *et al*., 2013).

Accounting for the functional and phylogenetic diversity of plant communities may thus be key to explaining the variation in herbivory along environmental gradients, in particular along gradients of decreasing plant species richness. This knowledge is of crucial importance in developing a better understanding of how biodiversity and its loss affect the impact of higher trophic levels on ecosystem functions. This is particularly relevant for species-rich subtropical and tropical forests, as they assume an important role in global biogeochemical cycles and climate regulation (Bonan, 2008), and for which the effects of herbivores are considered to be key modifiers of ecosystem processes (Schemske *et al*., 2009). Interestingly, although current theory on herbivore effects often emphasizes the role of specialists (see Cardinale *et al*., 2012), there is evidence that the impact of generalist herbivores can prevail over and differ from that of specialists in such highly diverse systems (Schuldt *et al*., 2010). Previous work in such forests has highlighted traits that might be particularly relevant in determining the overall differences in herbivory levels among woody plant species (Schuldt *et al*., 2012). However, so far, no study has attempted to mechanistically relate changes in species-specific herbivore damage with increasing woody plant diversity to functional trait and phylogenetic information of species-rich woody plant communities.

Here, we analyze to what extent functional and phylogenetic aspects of woody plant community composition contribute to improving our understanding of the role of biodiversity for herbivory patterns in highly diverse ecosystems. Our analysis builds on, and mechanistically extends, previous findings of increasing levels of herbivore damage on individuals of dominant tree and shrub species with increasing woody plant species richness in a subtropical forest system (Schuldt *et al*., 2010), and a particular focus of our study is on the performance of functional and phylogenetic diversity measures in explaining herbivory patterns relative to species richness effects. Effects of the former are usually not simply a reflection of the latter (Mason *et al*., 2008; Devictor *et al*., 2010). We study the relative effects of morphological and chemical leaf traits commonly considered to affect herbivory and the impact of woody plant phylogenetic diversity on species-specific herbivory levels across 27 forest stands in south-east China. We account for effects of community-weighted means (CWMs), trait diversity (based on single and multiple traits) and phylogenetic diversity, as well as of species-specific diversity measures. The relative impact of these different facets of community composition and diversity on ecosystem functions is only poorly known in natural systems (Mouillot *et al*., 2011). By focusing on these community-level measures, our approach takes into account the major sources of trait variation in these forest stands as, compared with the strong effects of interspecific variation, intraspecific trait variation within species has been found previously to play a very minor role in trait–environment relationships across the 27 study plots (Kröber *et al*., 2012). We hypothesize the following: that both functional and phylogenetic community metrics will explain the individual-level variation in observed herbivory better than will woody plant species richness; that not only individual traits, but multivariate diversity indices that combine the interactive effects of different traits, will be important predictors; and that, unlike in systems with specialized herbivore communities, the expected dominance of generalist herbivores in our study system (see Schuldt *et al*., 2010, 2012) is likely to promote positive interactions between herbivory and functional and phylogenetic diversity – which would be in contrast with predictions of general ecological theory for such highly diverse forests (see also Novotny *et al*., 2012).

## Materials and Methods

### Study site and herbivory assessment

The study was conducted in the Gutianshan National Nature Reserve (29°14′N, 118°07′E) in south-east China. The reserve covers *c*. 80 km² of evergreen mixed broadleaved forest, with *Castanopsis eyrei* and *Schima superba* as dominant tree species. The subtropical monsoon climate is characterized by a mean annual temperature of 15.3°C and a mean annual precipitation of *c*. 2000 mm (Hu & Yu, 2008). Within the reserve, 27 study plots of 30 × 30 m^2^ were established in 2008. The plots were selected to represent the range of woody plant species richness (25–69 tree and shrub species per plot) and successional stages (< 20–> 80 yr) found in the reserve (Bruelheide *et al*., 2011).

Herbivory was assessed on saplings (20–100 cm in height) of 10 dominant tree and shrub species: *Ardisia crenata* Sims, *Camellia fraterna* Hance, *Castanopsis eyrei* (Champ. ex Benth.) Tutch., *Cyclobalanopsis glauca* (Thunb.) Oerst., *Eurya muricata* Dunn, *Lithocarpus glaber* (Thunb.) Nakai, *Loropetalum chinense* (R. Br.) Oliv., *Machilus thunbergii* Sieb. et Zucc., *Neolitsea aurata* (Hayata) Koidz. and *Schima superba* Gardn. et Champ. These 10 evergreen species accounted for *c*. 50% of the total biomass of the tree and shrub layers in the study plots (see Schuldt *et al*., 2010). A maximum of 10 saplings per species and plot were checked for herbivory. Herbivory was quantified as the overall leaf damage caused by chewing, mining, galling and (if visible) sucking insects on all leaves of the saplings (mean number of leaves per sapling = 45.4 ± 45.3 SD). Assessments were conducted at the end of the rainy season in June/July 2008, which also marks the end of a major activity period for arthropods in these forests (Schuldt *et al*., 2012). We used predefined percentage classes (estimated as 0%, < 1%, 1–5%, > 5–15%, > 15–35% and > 35%; see, for example, Scherber *et al*., 2010; Schuldt *et al*., 2010; Ness *et al*., 2011) to visually assess standing levels of leaf damage. The actual, mean amount of damage for each estimated percentage class was then checked in detail by analyzing samples of randomly collected leaves (20–30) for each class; these were digitally scanned to determine the exact amount of leaf damage as the ratio of removed to estimated total leaf area (Schuldt *et al*., 2010, 2012). For the statistical analyses, we then used the mean damage of the scanned leaves of each class to calculate mean damage levels for each sapling (i.e. to account for potential deviations in the visually estimated damage from the digitally verified mean damage levels; for details, see Schuldt *et al*., 2010).

### Plant community data and general plot characteristics

For our analyses, we used a set of three morphological and four chemical leaf traits that are related to leaf quality and palatability, and that might thus particularly strongly affect herbivory (Coley & Barone, 1996; Perez-Harguindeguy *et al*., 2003; Poorter *et al*., 2004): leaf area (LA), specific leaf area (SLA) and leaf dry matter content (LDMC), as well as leaf C content, leaf C : N ratio, leaf C : P ratio and leaf polyphenolics content. The traits were measured for *c*. 80% of the 147 woody plant species recorded on the 27 study plots, and these species represented 95% of the total number of tree and shrub individuals at the study sites. As we used abundance-weighted indices to quantify functional community composition and diversity, these data should not be affected by the 5% of woody plant individuals for which trait values were missing. Data on leaf toughness, which has been shown in previous studies to potentially affect herbivory (Kitajima & Poorter, 2010), were only available for one-third of all species, and thus were not included in the analysis. However, Schuldt *et al*. (2012) showed that leaf toughness is probably not a limiting factor to herbivore damage in our study system. Details on trait measurements are provided in Kröber *et al*. (2012). In short, samples for trait measurements were taken from sun-exposed leaves of five to seven plant individuals in total, collected from up to seven plots per species in the summer of 2008. Trait measurements followed the standardized protocols of Cornelissen *et al*. (2003) and, for leaf polyphenolics, Hagermann (1987) (see Kröber *et al*., 2012). Our analysis focused on interspecific variation in trait values that determine community-level trait diversity, as intraspecific trait variability within species has been shown previously to have negligible effects on trait–environment relationships across our study plots (Kröber *et al*., 2012). Moreover, we show below that plot-level characteristics that can be expected to particularly strongly affect intraspecific trait variation (stand age, elevation and other abiotic conditions) were not retained in our final explanatory model, which further indicates that, unlike community-level trait diversity, intraspecific trait variation within species plays only a minor role in species-level variation in herbivory across the 27 study plots.

Phylogenetic data were obtained from an ultrametric phylogenetic tree of all angiosperm woody species recorded in the 27 study plots (Michalski & Durka, 2013). Woody plant species richness was recorded at the time of plot establishment in 2008 and was based on a complete inventory of all tree and shrub individuals of a height > 1 m (Bruelheide *et al*., 2011).

We also accounted for general plot characteristics, such as stand age, tree density, canopy cover, herb cover, elevation and aspect (see Bruelheide *et al*., 2011), as they might potentially confound diversity-functioning relationships in observational studies. Many of these characteristics were strongly correlated with each other, and we used principal components analysis (PCA) on these variables to obtain orthogonal predictor axes (for details of this analysis, see Schuldt *et al*., 2010). Only the first principal component axis (PC1_abio_), which represented stand age and age-dependent aspects of stand structure and biomass, was related to herbivore damage (Schuldt *et al*., 2010), and therefore was included in our analyses to account for diversity-independent plot effects. Other plot characteristics, as well as sapling height and the total number of saplings sampled, were shown by Schuldt *et al*. (2010) to have no effect on herbivory patterns of the study species.

### Diversity metrics and statistical analysis

In many cases, it remains unclear whether ecological functions are more strongly affected by CWM trait values, the variability within single traits or the diversity of multiple traits (Butterfield & Suding, 2013; Dias *et al*., 2013), and to what extent phylogenetic diversity provides additional information (Cadotte *et al*., 2009). To quantify the functional and phylogenetic aspects of the woody plant communities, we thus used a three-fold approach calculating: (1) Rao’s quadratic entropy *Q* (Rao, 1982) to assess plot-level trait and phylogenetic diversity; (2) CWM trait values to identify mass ratio effects of single traits; and (3) functional and phylogenetic relatedness between each of our focal species and all other species in the study plots to measure species-specific diversity effects.

Rao’s *Q* is calculated as the variance in pairwise dissimilarities among all individuals in a community. It can easily be applied to both functional and phylogenetic data, calculated for single as well as multiple traits, and weighted by abundance data (Schleuter *et al*., 2010; Pavoine & Bonsall, 2011). It thus enables a comparison between different facets of diversity using a consistent statistical framework (Pavoine & Bonsall, 2011). Moreover, as a measure of trait dispersion, Rao’s *Q* complements measures of CWM trait values (Ricotta & Moretti, 2011). Whereas CWM quantifies a community’s average functional trait value, weighted by the relative abundances of all individuals in this community, Rao’s *Q* provides a measure of trait variation around this mean. We calculated both CWM values and Rao’s *Q* for single traits (CWM_single.trait_, *Q*_single.trait_), as well as two multivariate versions of Rao’s *Q* that assessed the overall diversity of morphological (*Q*_morph_) and chemical (*Q*_chem_) leaf traits. We also tested for the effects of an overall Rao’s *Q* measure that integrates both the leaf morphological and chemical traits, but, as this measure was less strongly related to herbivory than was *Q*_chem_, we kept the distinction between morphological and chemical leaf trait diversity to allow for a better mechanistic interpretation of potential effects (although traits such as LDMC and C content might be related to some extent by both influencing leaf palatability (Poorter *et al*., 2009), the former also includes a strong morphological component (Kitajima & Poorter, 2010), and distinguishing between these effects via morphological and chemical trait diversity yielded straightforward results). Calculations of Rao’s *Q* were based on standardized trait values (mean = 0, SD = 1) and a Euclidean species distance matrix. For the multivariate measures of Rao’s *Q* based on the three morphological and four chemical traits, we used all axes of a PCA (as these axes are orthogonal to each other) on the standardized traits for the distance matrix to avoid collinearity effects (Böhnke *et al*., 2013; Purschke *et al*., 2013). For the phylogenetic data, we correspondingly calculated Rao’s *Q* from a phylogenetic cophenetic distance matrix (*Q*_phylo_). All measures of functional and phylogenetic diversity were weighted by plot-level abundance data to account for the relative impact of dominant vs rare species on community-level metrics.

In each plot, and for each of the 10 focal species, we further calculated a species-specific phylogenetic distance measure (*Q*^spec^_phylo_), based on the mean phylogenetic distance between an individual of a given focal species and all other woody plant individuals in a given study plot (Webb *et al*., 2002, 2006) – for consistency, we again expressed this measure as Rao’s *Q*, which, in the abundance-weighted case, is analogous to the MPD (mean phylogenetic distance) used in other studies (Vellend *et al*., 2011). Recent studies have shown that not only the overall phylogenetic diversity, but, in particular, the phylogenetic distance of a focal individual to all other individuals in a community, can determine herbivore effects (Webb *et al*., 2006; Paine *et al*., 2012; Parker *et al*., 2012). The species-specific measure of Rao’s *Q* was also calculated for trait data, and we included both multivariate relatedness measures for our focal species based on morphological (*Q*^spec^_morph_) and chemical (*Q*^spec^_chem_) leaf traits and measures for each individual trait (*Q*^spec^_T_, where T is the respective trait) in our analysis. Species-specific indices were calculated from the same distance matrices as used for the calculation of plot-level Rao’s *Q*, but by contrasting individuals of the respective focal species to all other individuals in each of the communities. Again, all measures were weighted by plot-level abundance data.

We used generalized linear mixed models with a binomial error structure (as a recommended way to analyze proportion data; Zuur *et al*., 2009), fitted by Laplace approximation (Bolker *et al*., 2009), to analyze the effects of functional and phylogenetic diversity metrics on the degree of herbivore damage of the 10 study species across the 27 study plots, whilst accounting for the effects of woody plant species richness and general plot characteristics. To determine which functional and phylogenetic characteristics particularly affect herbivory, and to assess whether their effects were complementary to simple species richness effects and independent of plot characteristics, we constructed five sets of models. These contained: (1) all predictors; (2) PC1_abio_ and all functional metrics (functional diversity *sensu* Diaz *et al*., 2007); (3) PC1_abio_ and phylogenetic metrics; (4) PC1_abio_ and woody plant species richness; and (5) only PC1_abio_. PC1_abio_ was included in all model sets to account for potentially confounding plot characteristics. Species identity, with individuals nested within species, and plot identity were considered as crossed random effects. The use of species identity as a random factor accounts for all interspecific differences in the levels of herbivory, leaving individual-level differences as the only source of variation. We also included a random factor with the total number of observations as factor levels to account for potential overdispersion in the data (Bates *et al*., 2013). Before the analysis, predictors were checked for collinearity and, where there was strong correlation (> 0.7) among predictors, we excluded those that were less strongly related to herbivory (e.g. CWM_C : N_ and CWM_C : P_, which were strongly correlated with CWM_Phenol_, but less strongly correlated with herbivory than CWM_Phenol_, and several correlated species-specific *Q*^spec^ measures; see Supporting Information Table S1 for a correlation matrix and a list of excluded variables). The final set of predictors included the general plot characteristics PC1_abio_, woody plant species richness, the phylogenetic diversity measure *Q*_phylo_, the multivariate chemical trait diversity *Q*_chem_, the single-trait dispersion variables *Q*_LDMC_, *Q*_C_, *Q*_C : N_, *Q*_Phenol_, the CWM values CWM_LA_, CWM_LDMC_, CWM_C_, CWM_Phenol_, and the species-specific measures *Q*^spec^_phylo_, *Q*^spec^_LA_, *Q*^spec^_LDMC_, *Q*^spec^_C_, *Q*^spec^_C : N_, *Q*^spec^_C : P_ and *Q*^spec^_Phenol_. We also included the interaction between woody plant species richness and overall phylogenetic diversity *Q*_phylo_, as this has been shown recently to influence species richness effects in grasslands (Dinnage, 2013). All predictors were standardized to a mean of zero and a standard deviation of unity before the analysis. Each model set was simplified by sequential deletion of predictors based on the reduction in the corrected Akaike information criterion (AICc) values to obtain the most parsimonious, minimal adequate model (which may potentially also contain variables that are not statistically significant at *P* < 0.05 if deletion of these variables would have markedly decreased the AICc fit; see Burnham & Anderson, 2004). The five resulting minimal adequate models were compared on the basis of their AICc values (ΔAICc) and AICc weights, with particularly low AICc values and high AICc weights indicating the best model fit (Burnham & Anderson, 2004). Model residuals were checked to comply with modeling assumptions. All analyses were performed with R 3.0.0 (http://www.R-project.org) and the package lme4 (Bates *et al*., 2013).

## Results

**Figure 1:**
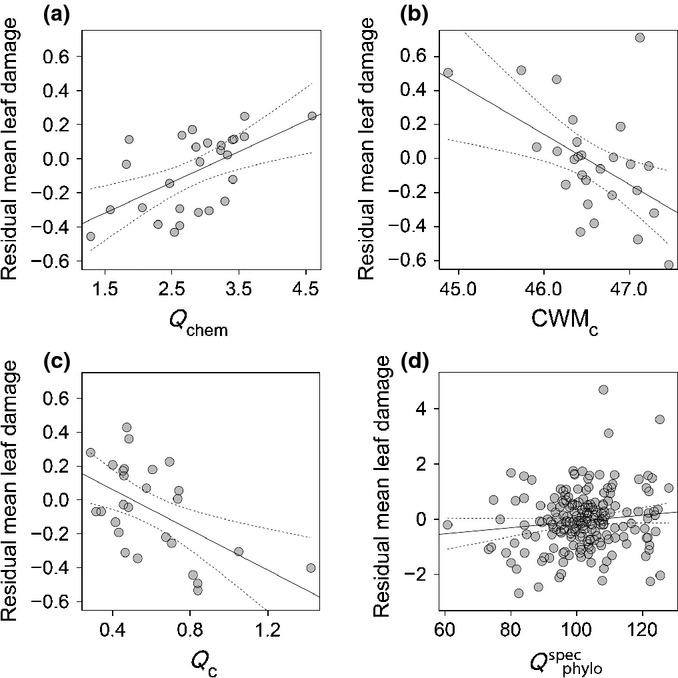
Independent effect on herbivore damage (partial residuals and 95% confidence bands) of (a) chemical leaf trait diversity (*Q*_chem_), (b) community-weighted mean leaf C values (CWM_C_), (c) leaf C content dispersion within the plant communities (*Q*_C_), and (d) species-specific mean phylogenetic distance of individuals of the target species to all other plant individuals in the plant communities; (a–c) show mean values of community-level measures across the 27 study plots, (d) shows mean values per study plot for each of the 10 target species. Standardized slopes are provided in Table1.

**Figure 2:**
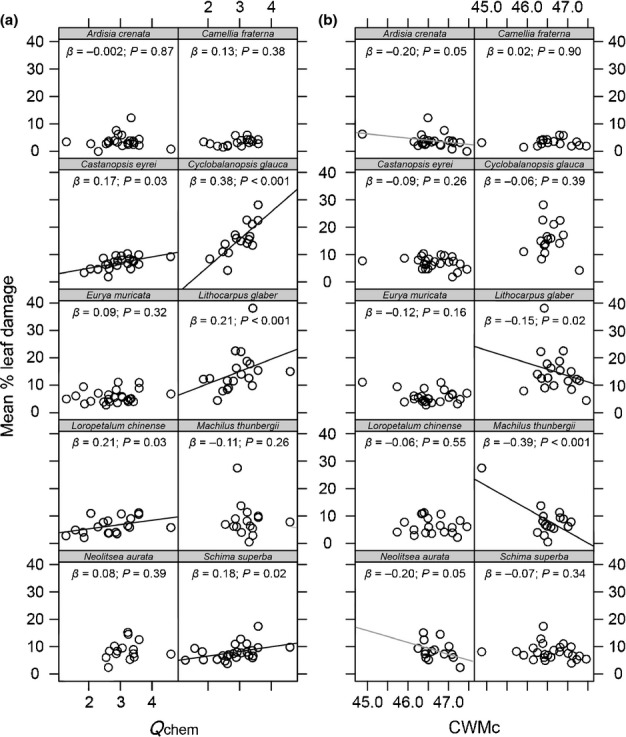
Relationships between herbivore damage of the single study species and (a) chemical leaf trait diversity (*Q*_chem_) and (b) community-weighted mean leaf C values (CWM_C_) (with regression slopes *β* and their probabilities *P*). Black lines indicate significant, gray lines close to significant relationships.

Mean leaf damage to the 10 study species, averaged across all 27 study plots, ranged between 3% (*Camellia fraterna*) and 17% (*Cyclobalanopsis glauca*). Species-specific damage levels varied by 15% (± 9.5% SD), on average, among the individual study plots. Species richness, functional characteristics and phylogenetic diversity of the plant communities all added essential explanatory value to the individual-level herbivory data. The minimal models based on abiotic characteristics and only phylogenetic or functional plant characteristics had a higher explanatory power than the models including only species richness and abiotic characteristics, or abiotic characteristics alone (Table1). By far the best minimal model with the highest empirical support (based on ΔAICc = 11.4 to the second-best model and an AICc weight of 1) was that derived from the full dataset. This model included woody plant species richness as well as a combination of functional and phylogenetic characteristics of the woody plant communities that were also included in the more simple functional and phylogenetic models (Table1). The multivariate Rao’s *Q* measure of chemical trait diversity (*Q*_chem_) and the CWM leaf C content of the plant communities (CWM_C_) contributed most to the overall best model, followed by weaker effects of woody plant species richness, the dispersion of leaf C content (*Q*_C_), the species-specific mean phylogenetic distance (*Q*^spec^_phylo_), the species-specific mean distance in LA (*Q*^spec^_LA_) and CWM_LDMC_ within the plant communities. It should be noted that the effects of most predictors were highly significant, and so potential issues of testing on the boundary of parameter space do not affect our results (Zuur *et al*., 2009). Herbivory decreased with increasing values of both CWM_C_ and *Q*_C_ (Fig.1b,c) and also of *Q*^spec^_LA_, whereas it was positively related to *Q*_chem_ and *Q*^spec^_phylo_ (Fig.1a,d), as well as to woody plant species richness and CWM_LDMC_. Abiotic plot characteristics were not included in the best minimal model (Table1), supporting our assumption that intraspecific trait variation promoted by these environmental characteristics was of little importance compared with community-level trait diversity. Single-regression relationships between herbivory and the two strongest predictors, *Q*_chem_ and CWM_C_, for the individual species show that the generalized relationships of the mixed model approach (although not statistically significant for all single species, but with a higher number of significant relationships than the one of 20 relationships expected by chance for *α* = 0.05) are well reflected in most of the individual species (Fig.2).

**Table 1:** Results for the fixed effects of the minimal generalized mixed-effects models on herbivore damage based on the full set of predictors and selected sets of predictors

Model	Fixed effects	Std. Est.	SE	*z*	*P*	AICc	ΔAICc	AICc_weight_
All predictors						996.6	0	1
	*Q*_chem_	0.19	0.04	5.1	< 0.0001			
	CWM_C_	−0.19	0.04	−4.9	< 0.0001			
	Woody plant species richness	0.14	0.04	3.9	0.0001			
	*Q*_C_	−0.14	0.04	−3.8	0.0002			
	*Q*^spec^_phylo_	0.14	0.04	3.0	0.0025			
	*Q*^spec^_LA_	−0.10	0.04	−2.4	0.0168			
	CWM_LDMC_	0.08	0.04	1.9	0.0529			
Functional structure + abiotic characteristics						1008.0	11.4	0
	*Q*_chem_	0.23	0.04	5.8	< 0.0001			
	*Q*_C_	−0.15	0.04	−3.6	0.0003			
	CWM_C_	−0.14	0.04	−4.0	0.0001			
	*Q*_LDMC_	0.07	0.04	1.9	0.0546			
Phylogenetic diversity + abiotic characteristics						1017.9	21.3	0
	PC1_abio_	0.19	0.05	3.7	0.0002			
	*Q*^spec^_phylo_	0.11	0.05	2.3	0.0198			
Species richness + abiotic characteristics						1019.6	23.0	0
	PC1_abio_	0.18	0.05	3.7	0.0002			
	Woody plant species richness	0.09	0.05	2.0	0.0492			
Abiotic characteristics only						1021.1	24.5	0
	PC1_abio_	0.19	0.05	3.8	0.0001			

Models are ordered by AICc, predictors within models by the absolute size of their standardized effects.

Std. Est, standardized slope; SE, standard error; AICc, corrected Akaike information criterion. Fixed effects in the minimal models are: Rao’s *Q* measures of leaf chemical trait diversity (*Q*_chem_), leaf C content dispersion (*Q*_C_), leaf dry matter content dispersion (*Q*_LDMC_), species-specific mean of phylogenetic distance of individuals of the target species to all other plant individuals in a community (*Q*^spec^_phylo_) and species-specific mean of leaf area trait dispersion (Q^spec^_LA_); community-weighted mean values of leaf C content (CWM_C_) and leaf dry matter content (CWM_LDMC_); woody plant species richness of the study plots; and the first principal component of a principal component analysis on general plot characteristics (PC1_abio_) that represents stand age and age-dependent aspects of stand structure and biomass.

## Discussion

Our study shows that measures of both functional and phylogenetic community characteristics contribute to explaining the variation in herbivory on tree recruits along a natural gradient in woody plant species richness – and that they clearly go beyond the explanatory power found previously for pure woody plant species richness in this respect (Schuldt *et al*., 2010). Our results particularly highlight the importance of multivariate trait variability, in addition to the effects of single traits, in informing our understanding of herbivory patterns in the context of biodiversity and ecosystem function relationships. Moreover, the positive relationships between herbivory and diversity measures contrast with common expectations for such highly diverse forests, and indicate that the way in which biodiversity affects the regulation of ecosystem functions requires a better understanding of the degree of food web specialization in such species-rich ecosystems.

### Trait interactions strongly affect herbivory

The best predictor of individual-level variation in herbivory across the 27 plots of our study was the multivariate *Q*_chem_, an integrative measure of the variation in leaf chemical traits (leaf C content, C : N and C : P ratios, leaf polyphenolics) which are considered to be of particular importance for the palatability of plants and their defense against herbivores (Coley & Barone, 1996; Perez-Harguindeguy *et al*., 2003; Poorter *et al*., 2004). Apparently, this multivariate index contains information that is not provided by single-trait measures of CWM values and variability. Several studies have shown that multivariate functional diversity indices can reveal non-additive effects that arise from interactions among species and traits (Mouillot *et al*., 2011; Dias *et al*., 2013). For herbivores, such interactions might encompass palatability and defense traits that determine trade-offs in resource use. This can become particularly relevant when multispecies assemblages of herbivores affect damage patterns: recent studies have shown that, under natural conditions, herbivory patterns are often much better explained by a complex of multiple traits (Agrawal & Fishbein, 2006; Carmona *et al*., 2011; Loranger *et al*., 2012; Schuldt *et al*., 2012). An interesting finding is that the traits represented in our *Q*_chem_ index appear to be less relevant in determining the general susceptibility of the studied plant species to herbivores than are, for instance, the morphological characteristics (Schuldt *et al*., 2012, but it should be noted that the latter study showed a positive relationship between leaf C content and LDMC – one of the strongest predictors of general susceptibility patterns among species in that study – such that palatability effects of the latter might be represented to some extent by the strong effects of C content in the present study). These leaf chemical traits may also often be of less relevance when only effects of trait variation within individual focal species are being considered (Carmona *et al*., 2011), rather than the effects of community-level trait variability on individual-level herbivory patterns (the latter of which was performed in the present study). A recent study in experimental grasslands highlighted the importance of such community effects by showing strong non-additive effects of species composition from monocultures to plant species mixtures on herbivore damage (Loranger *et al*., 2013). Thus, although the general susceptibility to herbivory may be strongly determined by the traits of a focal species (Schuldt *et al*., 2012), the trait composition (and, in part, traits other than those affecting mean herbivory susceptibility) of the surrounding plant community may become important in influencing the variation around these mean damage levels along environmental gradients (Barbosa *et al*., 2009). Recent findings of functionally more diverse diets of generalist (see below) herbivores in more diverse plant communities support this conclusion (Ibanez *et al*., 2013). The quantification of the relative impact of these effects is beyond the scope of our study and requires experimental manipulation (see Loranger *et al*., 2013). Yet, community-level trait metrics have also been identified as major drivers of ecosystem functions in many other studies (Butterfield & Suding, 2013; and references therein), indicating that they generally also affect species-specific patterns. In our case, the degree of herbivore damage of the study species among plots was positively related to the community-level diversity of leaf chemical traits – a pattern that does not necessarily match common predictions of general diversity–herbivory relationships (see Cardinale *et al*., 2012). This can be explained by the fact that many of the dominant herbivores in our study system are probably generalists that are not restricted to single host plant genera or families (Schuldt *et al*., 2010; M. Noack, A. Schuldt, T. Assmann, unpublished, showing that DNA-barcoded caterpillars of dominant Geometridae species were found on tree and shrub species belonging to more than one plant family). These herbivores can benefit from increased community-level variability of both palatability and defense traits, as this allows for complementary resource use and dietary mixing of host plants that differ in individual nutrient or defense characteristics (Pfisterer *et al*., 2003; Jactel & Brockerhoff, 2007; Schuldt *et al*., 2010).

### Single-trait measures complement multivariate indices in explaining herbivory

Effects of dietary mixing could also underlie the negative relationship between herbivory and the CWM levels of leaf C content (CWM_C_). The study species belonged to the tree and shrub species with a relatively high leaf C content (mean C content of the 10 study species was 47.8 ± 2.5% SD, compared with a range between 35% and 51% for the remaining species in the communities and a maximum CWM_C_ observed for our study plots of 47.5%). Herbivore damage on these species might decline if increasing CWM_C_ decreases the probability of herbivores being able to use alternative host plants with lower leaf C content (which are more abundant in low CWM_C_ communities) to compensate for low nutrient quality in their preferred hosts (potentially a mix of different nutrients, as indicated by the strong *Q*_chem_ effect and the absence of C : N or C : P metrics in the minimal models (or of phenolic content, with which these ratios were, in part, strongly correlated and thus not included directly in the models)). We might also potentially have expected an effect of the species-specific *Q*^spec^_C_ in this case. However, the fact that this variable did not provide additional explanation could be because nutrient quality effects are largely captured by the more integrative *Q*_chem_, with additional variation already largely explained by the effects of CWM_C_ and *Q*_C_.

Effects of the variability in leaf C content (*Q*_C_) on herbivory might be explained by interrelations with CWM_C_ (see also Ricotta & Moretti, 2011; Dias *et al*., 2013 for interaction effects between CWM and trait variability). Low *Q*_C_ can apply to both communities with overall high, but also overall low, leaf C content of the constituent species. In our study, the communities with low *Q*_C_ tended to have a lower rather than higher CWM_C_ (Pearson’s *r* = 0.3; *P* = 0.12, see Table S1), such that low community-level variability in leaf C content could indicate better nutrient conditions. However, such a relationship would only be moderate in our case, as adding an interaction term for *Q*_C_ and CWM_C_ did not improve the model fit (which could be explained by the fact that low *Q*_C_ and CWM_C_ only coincide at low leaf C concentrations, whereas high CWM_C_ might display both high and low variation in leaf C contents).

### Phylogenetic relatedness is more important than overall phylogenetic diversity

In contrast with leaf chemical traits, phylogenetic diversity measures were of less importance in explaining variation in herbivory across the 27 study plots (and, for our system, we were unable to detect an interaction between phylogenetic diversity and plant species richness, as recently reported by Dinnage (2013) for grasslands). This was not caused by potential phylogenetic clustering in functional traits masking actual phylogenetic effects, as the model fit for phylogenetic data was low even when considered in isolation of functional traits (ΔAICc = 9.9 compared with the minimal model based on functional traits; Table1). However, although the overall phylogenetic diversity of the woody plant communities had little effect (*Q*_phylo_ was not included in the best overall model or in the minimal phylogenetic model), herbivory was positively related to the species-specific measure *Q*^spec^_phylo_. As also indicated by the results for CWM_C_, this makes it clear that the position of a focal species within trait space (in the case of *Q*^spec^_phylo_ approximated by a phylogenetic measure) can provide information that is not captured by, and not necessarily dependent on, overall community diversity (Butterfield & Suding, 2013). The positive effect of *Q*^spec^_phylo_ is contrary to the effects reported for similar measures from other species-rich forests, where phylogenetic diversity and relatedness have been observed to decrease species-specific levels of herbivory via mechanisms of negative density dependence (Webb *et al*., 2006; Ness *et al*., 2011; Paine *et al*., 2012). Yet, the positive effect is congruent with our findings for overall leaf chemical diversity and the expected impact of generalist herbivores (see also Parker *et al*., 2012; Castagneyrol *et al*., 2013). It thus supports our expectation that feeding specialization strongly determines how consumers affect the relationship between biodiversity and ecosystem functions (Thebault & Loreau, 2003; Cavender-Bares *et al*., 2009).

### Species richness provides additional information

Although functional trait and phylogenetic information outperformed pure woody plant species richness in explaining the variability in herbivore levels across the 27 study plots, species richness was nevertheless retained as a predictor in the best minimal model (for a detailed discussion of the relationship between species richness and herbivory in our study system, see Schuldt *et al*., 2010). Although mechanistically advancing our understanding of diversity effects on herbivory compared with the analysis considering only species richness (Schuldt *et al*., 2010), our measures of trait diversity and also the inclusion of phylogenetic diversity apparently do not fully account for the information contained in the simple species richness measure. This might indicate the effects of unmeasured traits that are not phylogenetically conserved, or interaction effects not captured by our multivariate diversity indices, and shows the limitations of phylogenetic measures as a surrogate measure of functional trait variation (Srivastava *et al*., 2012).

### Community-level consequences

The patterns we observed are likely to result in negative effects on the growth of our study species, as even low levels of persistent herbivore damage can strongly decrease plant fitness (Zvereva *et al*., 2012). Our study species belong to the dominant woody plants in our study system, and increasing damage with increasing plant diversity might potentially promote overall woody plant diversity (but note that we lack long-term data from our study system). As the growth of tree and shrub recruits determines woody plant diversity in the long term, we would expect negative effects on diversity if all woody plant species were equally affected by herbivory. In particular, the effects of *Q*_chem_ and *Q*_phylo_ could potentially promote clustering over time in the phylogenetic composition and the trait space occupied by the woody plant communities (see also Cavender-Bares *et al*., 2009). However, these effects will be mediated by eco-evolutionary feedbacks between plant and herbivore communities, with changes in plant communities affecting herbivores and their impact on plants, plant trait composition and diversity (Johnson *et al*., 2009; Carmona & Fornoni, 2013). Such feedbacks can result in dynamic processes that require longer term data for a better understanding of the complex interactions between herbivores and their hosts. The observed high plant species and functional diversity in the natural forests of our study suggest either that the benefits of increased functional diversity (e.g. better resource partitioning among plants; Cardinale *et al*., 2012) outweigh the negative effects of herbivory or that not all species show the positive diversity–herbivory relationship. Several studies have suggested that abundant and rare species can be affected by herbivory in contrasting ways, resulting in a community compensatory trend that stabilizes diversity (Queensborough *et al*., 2007; Chen *et al*., 2010). High functional diversity could thus be maintained by less abundant species that profit from increased herbivory of abundant species – and a potentially lower fitness and reduced impact of these species on other species – under these conditions. The fact that abundant woody plant species at our study site have been found previously to experience higher mean damage levels than less common species supports this assumption (see Schuldt *et al*., 2012).

### Conclusions

Our study shows how a combined approach that incorporates different facets of functional and phylogenetic community composition and diversity can help in informing our mechanistic understanding of how biodiversity affects ecosystem functions along natural environmental gradients. It emphasizes the impact of community-level functional properties on a set of focal species, which deviates from previously reported effects of species-specific trait variation within and among these species. Considering that individual species usually form part of larger communities (see also Karban, 2010), these community effects can help to better predict biodiversity–ecosystem function relationships under changing environmental conditions. Species richness, although mechanistically less informative, can add to this framework by indicating effects of unmeasured traits that are not phylogenetically conserved or interactive effects of traits that are not captured by multivariate diversity indices. With increasing loss of species, but, in particular, with the concomitant loss of functional variability and phylogenetic information in a community, the impact of herbivores can be expected to change – with consequences for the herbivore-mediated regulation of ecosystem functions and properties. In this respect, the largely positive relationship between herbivory and different facets of diversity indicates that the degree of food web specialization within a community is of crucial significance for the way in which biodiversity loss will affect ecosystem functioning.

## Supporting Information

**Table S1** Correlation matrix of predictors
