# Supplementary material for: Functional and phylogenetic diversity of woody plants drive herbivory in a highly diverse forest
Source: New Phytol. 2014 Jan 24;202(3):864–73. doi: 10.1111/nph.12695 (PMC4235298; doi:10.1111/nph.12695)
Supplement: Supplementary file 1 — Table S1 Correlation matrix of predictors [file nph0202-0864-SD1.docx]

**Supporting information**

**Table S1** Correlation matrix for plot means of the response variable (herbivory) and the predictor variables considered in the study. Variables not included in the full model due to strong correlation (>0.7, printed in bold) with other predictors (the latter of which, in turn, are more strongly correlated with the response variable than the dropped predictors) are: Q_morph_, Q_LA_, Q_SLA_, Q_CP_, CWM_SLA_, CWM_CN_, CWM_CP_, *Q*^spec^_chem_, *Q*^spec^_morph_, *Q*^spec^_SLA_

|  | Herbivory | Richness | PC1 | Q_phylo_ | Q_chem_ | Q_morph_ | CWM_LA_ | CWM_SLA_ | CWM_LDMC_ | CWM_C_ | CWM_C:N_ | CWM_C:P_ | CWM_Phenol_ | Q_LA_ | Q_SLA_ | Q_LDMC_ | Q_C_ | Q_C:N_ | Q_C:P_ | Q_phenol_ | Q^spec^_morph_ | Q^spec^_chem_ | Q^spec^_LA_ | Q^spec^_SLA_ | Q^spec^_LDMC_ | Q^spec^_C_ | Q^spec^_C:N_ | Q^spec^_C:P_ | Q^spec^_Phenol_ |
| --- | --- | --- | --- | --- | --- | --- | --- | --- | --- | --- | --- | --- | --- | --- | --- | --- | --- | --- | --- | --- | --- | --- | --- | --- | --- | --- | --- | --- | --- |
| Richness | 0.47 |  |  |  |  |  |  |  |  |  |  |  |  |  |  |  |  |  |  |  |  |  |  |  |  |  |  |  |  |
| PC1 | 0.65 | 0.19 |  |  |  |  |  |  |  |  |  |  |  |  |  |  |  |  |  |  |  |  |  |  |  |  |  |  |  |
| Q_phylo_ | 0.36 | 0.48 | 0.42 |  |  |  |  |  |  |  |  |  |  |  |  |  |  |  |  |  |  |  |  |  |  |  |  |  |  |
| Q_chem_ | 0.57 | 0.35 | 0.63 | 0.64 |  |  |  |  |  |  |  |  |  |  |  |  |  |  |  |  |  |  |  |  |  |  |  |  |  |
| Q_morph_ | -0.08 | 0.18 | -0.29 | -0.32 | -0.30 |  |  |  |  |  |  |  |  |  |  |  |  |  |  |  |  |  |  |  |  |  |  |  |  |
| CWM_LA_ | 0.15 | 0.56 | -0.05 | -0.05 | -0.10 | **0.73** |  |  |  |  |  |  |  |  |  |  |  |  |  |  |  |  |  |  |  |  |  |  |  |
| CWM_SLA_ | -0.28 | -0.25 | -0.47 | -0.45 | -0.63 | 0.68 | 0.25 |  |  |  |  |  |  |  |  |  |  |  |  |  |  |  |  |  |  |  |  |  |  |
| CWM_LDMC_ | -0.25 | -0.07 | 0.11 | 0.07 | 0.11 | -0.30 | -0.22 | -0.48 |  |  |  |  |  |  |  |  |  |  |  |  |  |  |  |  |  |  |  |  |  |
| CWM_C_ | -0.35 | 0.09 | -0.04 | 0.44 | 0.20 | -0.16 | -0.06 | -0.30 | 0.58 |  |  |  |  |  |  |  |  |  |  |  |  |  |  |  |  |  |  |  |  |
| CWM_C:N_ | 0.40 | 0.25 | 0.53 | 0.57 | 0.68 | -0.68 | -0.26 | -**0.93** | 0.25 | 0.26 |  |  |  |  |  |  |  |  |  |  |  |  |  |  |  |  |  |  |  |
| CWM_C:P_ | 0.47 | 0.27 | 0.62 | 0.52 | **0.80** | -0.47 | -0.09 | -**0.80** | 0.16 | 0.31 | **0.88** |  |  |  |  |  |  |  |  |  |  |  |  |  |  |  |  |  |  |
| CWM_Phenol_ | 0.55 | 0.43 | 0.52 | 0.43 | 0.69 | -0.40 | 0.10 | -**0.82** | 0.09 | 0.07 | **0.85** | **0.81** |  |  |  |  |  |  |  |  |  |  |  |  |  |  |  |  |  |
| Q_LA_ | -0.05 | 0.18 | -0.34 | -0.23 | -0.31 | **0.86** | **0.74** | 0.61 | -0.42 | -0.17 | -0.56 | -0.43 | -0.28 |  |  |  |  |  |  |  |  |  |  |  |  |  |  |  |  |
| Q_SLA_ | -0.12 | -0.03 | -0.28 | -0.47 | -0.43 | **0.83** | 0.48 | **0.81** | -0.42 | -0.23 | -**0.74** | -0.49 | -0.54 | 0.61 |  |  |  |  |  |  |  |  |  |  |  |  |  |  |  |
| Q_LDMC_ | 0.00 | 0.24 | 0.11 | 0.05 | 0.20 | 0.37 | 0.23 | -0.08 | 0.38 | 0.12 | -0.09 | 0.00 | -0.02 | -0.05 | 0.12 |  |  |  |  |  |  |  |  |  |  |  |  |  |  |
| Q_C_ | -0.14 | 0.07 | 0.11 | 0.24 | 0.50 | -0.13 | -0.25 | -0.39 | 0.27 | 0.30 | 0.29 | 0.42 | 0.13 | -0.37 | -0.18 | 0.51 |  |  |  |  |  |  |  |  |  |  |  |  |  |
| Q_C:N_ | 0.62 | 0.40 | 0.46 | 0.18 | 0.52 | 0.18 | 0.25 | -0.17 | -0.18 | -0.18 | 0.30 | 0.43 | 0.38 | 0.11 | 0.20 | 0.10 | 0.11 |  |  |  |  |  |  |  |  |  |  |  |  |
| Q_C:P_ | 0.33 | 0.02 | 0.54 | 0.48 | **0.71** | -0.46 | -0.26 | -0.52 | 0.26 | 0.38 | 0.58 | 0.66 | 0.42 | -0.28 | -0.54 | -0.20 | 0.18 | 0.24 |  |  |  |  |  |  |  |  |  |  |  |
| Q_phenol_ | 0.55 | 0.45 | 0.32 | 0.44 | 0.55 | -0.02 | 0.19 | -0.27 | -0.20 | -0.19 | 0.31 | 0.32 | 0.59 | -0.07 | -0.16 | 0.24 | 0.04 | 0.24 | -0.04 |  |  |  |  |  |  |  |  |  |  |
| Q^spec^_morph_ | 0.11 | 0.07 | -0.10 | -0.12 | -0.13 | 0.52 | 0.33 | 0.45 | -0.21 | -0.06 | -0.43 | -0.23 | -0.27 | 0.39 | 0.50 | 0.22 | -0.01 | 0.09 | -0.22 | 0.01 |  |  |  |  |  |  |  |  |  |
| Q^spec^_chem_ | -0.09 | 0.07 | 0.10 | 0.03 | 0.15 | 0.01 | 0.05 | -0.04 | -0.03 | -0.09 | 0.04 | 0.06 | 0.10 | 0.00 | -0.02 | 0.07 | 0.01 | 0.12 | 0.11 | 0.09 | 0.17 |  |  |  |  |  |  |  |  |
| Q^spec^_phylo_ | 0.05 | 0.04 | 0.15 | 0.15 | 0.16 | 0.01 | 0.03 | 0.02 | -0.04 | 0.02 | 0.01 | 0.09 | -0.01 | -0.02 | 0.02 | 0.07 | 0.08 | 0.12 | 0.13 | 0.03 | 0.39 | 0.35 |  |  |  |  |  |  |  |
| Q^spec^_LA_ | 0.10 | 0.32 | -0.19 | -0.01 | -0.21 | 0.65 | 0.64 | 0.42 | -0.20 | 0.00 | -0.43 | -0.29 | -0.17 | **0.72** | 0.39 | 0.12 | -0.26 | -0.03 | -0.23 | 0.07 | 0.23 | -0.11 | -0.08 |  |  |  |  |  |  |
| Q^spec^_SLA_ | -0.09 | -0.11 | -0.32 | -0.38 | -0.42 | 0.62 | 0.25 | **0.81** | -0.41 | -0.21 | **-0.75** | -0.53 | -0.60 | 0.47 | **0.76** | 0.04 | -0.20 | 0.05 | -0.41 | -0.14 | 0.46 | 0.03 | 0.08 | 0.41 |  |  |  |  |  |
| Q^spec^_LDMC_ | 0.22 | 0.07 | 0.11 | 0.09 | 0.13 | 0.09 | 0.07 | -0.02 | -0.01 | 0.04 | 0.02 | 0.10 | 0.05 | 0.01 | 0.07 | 0.18 | 0.16 | 0.09 | 0.01 | 0.09 | **0.81** | 0.14 | 0.41 | -0.14 | -0.10 |  |  |  |  |
| Q^spec^_C_ | -0.13 | 0.00 | -0.03 | -0.08 | 0.02 | 0.01 | 0.01 | -0.03 | -0.05 | -0.11 | 0.02 | 0.02 | 0.04 | -0.03 | 0.02 | 0.06 | 0.08 | 0.07 | -0.07 | 0.04 | 0.14 | 0.43 | 0.16 | -0.19 | 0.01 | 0.14 |  |  |  |
| Q^spec^_C:N_ | -0.11 | -0.04 | 0.11 | -0.23 | -0.03 | 0.18 | 0.13 | 0.20 | -0.09 | -0.23 | -0.18 | -0.08 | -0.13 | 0.12 | 0.25 | 0.01 | -0.12 | 0.32 | 0.04 | -0.17 | 0.16 | 0.40 | 0.26 | -0.12 | 0.39 | -0.07 | 0.43 |  |  |
| Q^spec^_C:P_ | 0.29 | 0.08 | 0.28 | 0.16 | 0.31 | -0.11 | -0.01 | -0.18 | 0.05 | 0.05 | 0.22 | 0.30 | 0.20 | -0.04 | -0.14 | -0.08 | 0.02 | 0.20 | 0.39 | -0.03 | -0.08 | 0.24 | 0.22 | 0.01 | -0.06 | -0.01 | -0.28 | 0.13 |  |
| Q^spec^_Phenol_ | -0.16 | 0.06 | -0.09 | 0.02 | -0.06 | 0.08 | 0.06 | 0.09 | -0.04 | -0.05 | -0.12 | -0.17 | -0.04 | 0.06 | 0.04 | 0.10 | -0.07 | -0.11 | -0.13 | 0.14 | 0.18 | **0.76** | 0.17 | 0.06 | 0.06 | 0.11 | 0.07 | -0.02 | -0.14 |
|  | Herbivory | Richness | PC1 | Q_phylo_ | Q_chem_ | Q_morph_ | CWM_LA_ | CWM_SLA_ | CWM_LDMC_ | CWM_C_ | CWM_C:N_ | CWM_C:P_ | CWM_Phenol_ | Q_LA_ | Q_SLA_ | Q_LDMC_ | Q_C_ | Q_C:N_ | Q_C:P_ | Q_phenol_ | Q^spec^_morph_ | Q^spec^_chem_ | Q^spec^_LA_ | Q^spec^_SLA_ | Q^spec^_LDMC_ | Q^spec^_C_ | Q^spec^_C:N_ | Q^spec^_C:P_ | Q^spec^_Phenol_ |

Abbreviations: Richness = Woody plant species richness; PC1 = first principal component of a PCA on general plot characteristics; Qphylo, Qchem, Qmorph = dissimilarity in phylogenetic (phyl), chemical trait (chem), and morphological (morph) trait diversity, respectively, of the woody plant communities; CWM_T_ = community weighted mean values, Q_T_ = plot level dispersion, Q^spec^_T_ = species-specific mean distance of individuals of the target species to all other plant individuals in a community for Trait T, where T = leaf area (LA), specific leaf area (SLA), leaf dry matter content (LDMC), leaf C content (C), leaf C:N ratio (C:N), leaf C:P ratio (C:P), and leaf phenolics content (Phenol).
